# Ultrasound and Microwave-Assisted Extraction of Blackberry (*Rubus fruticosus* L.) Pomace: Analysis of Chemical Properties and Anticancer Activity

**DOI:** 10.3390/plants14030384

**Published:** 2025-01-27

**Authors:** Indrė Čechovičienė, Živilė Tarasevičienė, Ewelina Hallman, Agata Jabłońska-Trypuć, Laima Česonienė, Daiva Šileikienė

**Affiliations:** 1 Department of Plant Biology and Food Sciences, Agriculture Academy Vytautas Magnus University, Donelaičio Str. 58, 44248 Kaunas, Lithuania; 2 Department of Functional and Organic Food, Institute of Human Nutrition Sciences, Warsaw University of Life Sciences, Nowoursynowska Str. 159C, 02-776 Warsaw, Poland; 3 Bioeconomy Research Institute, Agriculture Academy, Vytautas Magnus University, Donelaičio Str. 52, LT-44248 Kaunas, Lithuania; 4 Department of Chemistry, Biology and Biotechnology, Faculty of Civil Engineering and Environmental Sciences, Bialystok University of Technology, 15-351 Białystok, Poland; 5 Department of Environment and Ecology, Agriculture Academy, Vytautas Magnus University, Donelaičio Str. 58, 44248 Kaunas, Lithuania

**Keywords:** pomace, phenolic profile, ultrasound-assisted extraction, microwave-assisted extraction, Caco-2 cells, CCD-18Co

## Abstract

Blackberries are seasonal berries that are processed into various products leaving a large amount of residues after processing, and therefore the most effective ways of utilising the residues need to be evaluated. The aim of this study was to determine the effect of different extraction methods on the chemical content of blackberry pomace extracts from different cultivars and their effect on the viability of the Caco-2 colorectal adenocarcinoma cell line and CCD-18Co normal colon fibroblast cancer cells. Blackberry pomace from berries of the cultivars ‘Polar’, ‘Orkan’, and ‘Brzezina’ was extracted by ultrasound-assisted extraction (UAE), microwave-assisted extraction (MAE) and a combination of these two extraction methods (MAE+UAE). The phenolic profile and the amount of organic acids and sugars were detected by HPLC. The spectrophotometric method was used to determine the amount of total phenolics, total flavonoids, and total anthocyanins. The cytotoxicity of the extracts was measured by the MTT assay. The chemical content of the extracts depends on the blackberry cultivar, the extraction method, and its interaction. Only the DPPH antioxidant activity did not depend on these factors and had no statistically significant differences between the different extracts. The extracts at a concentration of 5.0% increased the growth of both cancer cells, while the extracts at 1% and 2.5%, depending on the cultivar, reduced the growth of these cells. The MAE and UAE extracts of the ‘Orkan’ cultivar at concentrations of 1%, 1.5%, 2%, and 2.5% best inhibited the viability of Caco-2 cells. The extracts inhibited the growth of the Caco-2 cell line better than CCD-18Co normal colon fibroblasts.

## 1. Introduction

The short postharvest life of blackberries is due to their high respiration rate and fragile structure, which limits their consumption when fresh. Therefore, blackberries are processed [1]. Blackberry pomace is a very important source of biologically active compounds [2] known for their antioxidant, anti-inflammatory, anticancer, antiviral, and cardiovascular effects [3]. This agro-food by-product is rich in polyphenolic compounds such as flavonoids (epigallocatechin, catechin, epigallocatechin gallate, quercetin-3-*O*-rutinoside, kaempferol-3-*O*-glucoside, myricetin, quercetin, kaempferol, quercetin-3-*O*-glucoside), phenolic acids (chlorogenic, ellagic, *p*-coumaric), and anthocyanins (cyanidin-3-*O*-glucoside and cyanidin-3-*O*-rutinoside) [4].

In addition to being a good source of biologically active compounds, blackberry pomace is also rich in dietary fibre and can be used as a functional food ingredient to improve the texture and stability of food products. Despite these facts, the direct use of blackberry pomace in food is not always the best option due to the insufficient phytochemical content and the negative and rapid environmental impact on its quality. Therefore, the use of pomace extracts may be a promising method to increase its utilisation possibilities [5] and increase the preservation of biologically active compounds.

Two advanced technologies, microwave-assisted and ultrasound-assisted extraction, are recognised as promising green extraction technologies [6] and have been successfully used to extract thermo-sensitive phytochemicals from many plant sources [5], including blackberries [6,7]. In order to produce a safe and environmentally friendly extracted product, the use of so-called GRAS (Generally Recognised as Safe) green solvents, such as ethanol, water, and mixtures of the two, is gaining increasing attention [8].

The advantages of ultrasound-assisted extraction (UAE) are its high efficiency, short extraction time, ability to extract at low temperatures, low solvent consumption, and good extract quality. During UAE, plant tissues are damaged (by erosion, pore formation, fragmentation, and other phenomena), and bioactive compounds may be released into the solvent [7]. According to Machado et al. [8], UAE was the most effective method to recover anthocyanins from blackberry pomace. Other researchers found that ultrasound-assisted extraction was the most effective method to extract bioactive compounds from blackberry residues [9], blueberries pomace [10,11], raspberry puree [12], and raspberry pomace [11]. According to Nastić et al. [13], ultrasound-assisted extraction provided the best results for extraction yield, total phenolics, and several compounds such as cyanidin-3-*O*-rutinoside, cyanidin-3-*O*-glucoside, rutin, ellagic acid, and gallic acid in black raspberry pomace extracts.

In microwave-assisted extraction (MAE), the sample is heated directly; as a result of the heat, the target compounds are extracted quickly and efficiently, and their solubility and diffusion are also increased [14]. According to Ferrara et al. [14], this is the most commonly used method for the extraction of phenolic compounds (phenolic acids, flavonoids, anthocyanins, and tannins). Carvalho et al. [15] argue that microwave-assisted extraction of phenolics can be efficient or not, depending on the solvents used and the experimental conditions (temperature and time). Blackberry extracts obtained by microwave-assisted extraction have higher total anthocyanin concentrations than those obtained by ultrasound-assisted extraction [6]. According to Belwal et al. [16], microwave extraction is a more efficient method than ultrasound extraction for extracting anthocyanins, polyphenols, and flavonoids from various plant materials. Teng et al. [17] reported that microwave-assisted extraction provided the highest extraction yield of these compounds: chlorogenic acid (2.50 mg g^−1^), syringic acid (2.17 mg g^−1^), rutin (1.89 mg g^−1^), (+)-catechin (1.42 mg g^−1^), gallic acid (0.63 mg g^−1^), *p*-coumaric acid (0.56 mg g^−1^), ferulic acid (0.50 mg g^−1^), quercetin (0.40 mg g^−1^), and caffeic acid (0.39 mg g^−1^) in raspberry extract. MAE is often combined with other techniques, such as UAE [18]. Combinations of these technologies (MAE+UAE) may be an option to achieve a better balance between product quality, production costs, and solvent use [19].

According to Gil-Martínez et al. [20], blackberry extracts can inhibit the proliferation of different types of colorectal tumours. The authors also indicate that this effect is due to the combined actions of several phytochemicals found naturally in blackberry fruit, which produced the anti-inflammatory effect shown by blackberry extract [20]. Četojević-Simin et al. [21] report that the synergistic effects of organic acids, phenolic acids, tannins, and other constituents are responsible for the antitumor activity of blackberry (‘Čačanska bestrna’ and ‘Thornfree’) pomace extracts.

Despite the existing research data on the influence of different extraction methods on the chemical composition of blackberry pomace extracts, there is a lack of knowledge on how the cultivar of blackberry and the combination of different extraction methods influence extraction efficiency, the biochemical composition of the extracts, and their effect on cancer cell viability.

## 2. Results and Discussion

### 2.1. Total Polyphenol, Anthocyanin, Flavonoid Concentrations, and Antioxidant Activities (DPPH^•^ and ABTS^•+^) in Blackberry Pomace Extracts

The total phenolic content (TPC), total anthocyanin content (TAC), and total flavonoid content (TFC) varied significantly (*p* < 0.05) depending on the cultivar, extraction method, and their interaction (Table 1). The general trends are noted for TPC and TFC, showing that the highest amount was observed in the UAE extract of the cultivar ‘Orkan’ with 3694.25 mg 100 g^−1^ and 239.88 mg 100 g^−1^ of soluble solids of extracts, respectively. Conversely, the lowest amount of these compounds was found in the UAE extract of cultivar ‘Brzezina’, with 2032.75 mg 100 g^−1^ and 93.53 mg 100 g^−1^ of soluble solids of extracts, respectively. However, the analysis of the data did not reveal any general trends in the extraction efficiency of the compounds for the different blackberry cultivars. With regards to the total phenolic compounds in the ‘Polar’ cultivar pomace extracts, there was no significant difference in their content in pomace extracts using all extraction methods (Table 1). In the pomace extract of the ‘Orkan’ cultivar, on the other hand, the highest total phenolic content was found in MAE and UAE, and in ‘Brzezina’—MAE.

For TFC, the combination of MAE and UAE was the most effective for the ‘Orkan’ and ‘Brzezina’ cultivar pomace extracts. According to Yu et al. [22], the interaction of the two methods, MAE and UAE, affects the maximum TFC. The authors also found that a higher TFC is found when microwaves are used first, followed by ultrasound (MAE+UAE), rather than the opposite (UAE+MAE). According to Mikucka et al. [23], microwave energy and sonic cavitation are the primary elements influencing the effectiveness of the recovery of all phenolic compounds in MAE and UAE, respectively. When cavitation bubbles build up to sufficiently high pressures during UAE, acoustic cavitation causes them to collapse or implode, releasing a significant amount of energy. At the same time, high shear forces and turbulence are generated around the cavitation bubbles.

The highest and lowest amounts of TAC were found in the MAE extract of the cultivar ‘Brzezina’—129.91 mg 100 g^−1^ of soluble solids of extracts, and the cultivar ‘Polar’—78.56 mg 100 g^−1^ of soluble solids of extracts, respectively.

Ultrasound-assisted extraction had the greatest effect on the total anthocyanin content of pomace extracts from the ‘Polar’ and ‘Orkan’ cultivars, while the opposite effect was observed in ‘Brezina’—MAE. According to dos Santos et al. [24], the optimal UAE conditions (ultrasound amplitude of 40% for 10 min and a concentration of 25 mg/L) resulted in the maximum total anthocyanin concentration in blackberry pomace extracts. According to Yu et al. [22], UAE-treated plant cell walls and membranes are more permeable to solvents (during cell tissue rupture) than those treated with MAE, as determined by scanning electron micrographs before and after treatment. During UAE, a specific process called cavitation occurs, which is the abrupt collapse of micro-bubbles created by pressure changes, allowing for the release of various compounds, including anthocyanins [25].

Several studies indicate that blackberry pomace extracts have a high antioxidant capacity, suggesting that they can scavenge free radicals and reduce oxidative stress, which has been linked to a number of chronic diseases [24,26]. Non-significant differences in DPPH assay values were obtained between all cultivars and extraction methods after statistical analysis, and values varied from 66.73 to 85.85 µmol TE g^−1^ depending on the cultivar and extraction method (Table 1).

The ABTS^•+^ antioxidant activity varied significantly depending on the cultivar and its interaction with the extraction method. The analysis of the results showed a tendency for the cultivars ‘Polar’ and ‘Brzezina’ to have the highest ABTS^•+^ values in the UAE extract, while ‘Orkan’—UAE+MAE. The antioxidant activity of the extracts can be reduced by hydrolysis conditions, which have a significant impact on the overall yield and profile of phenolic acids [23,27]. The UAE extract of the ‘Polar’ cultivar showed the highest radical scavenging activity, whereas the UAE extract of the ‘Orkan’ cultivar showed the lowest activity according to the ABTS^•+^ assay.

The antioxidant activity determined by the ABTS^•+^ assay ranged from 303.80 μmol TE g^−1^ to 171.16 μmol TE g^−1^ soluble solids of extracts, which was significantly higher than that determined by the DPPH^•^ assay (Table 1). The antioxidant activity may depend on the extraction solvent and the phenolic compounds, as different phenolic compounds react differently in different antioxidant activity assays [28]. According to Szymanowska et al. [29], the high antioxidant activity of blackberry pomace extracts is mainly due to its anthocyanin content, which is associated with an increased ABTS^•+^ scavenging capacity. The properties of specific compounds, such as flavonoids and anthocyanins, and the number and position of hydroxyl groups, may enhance their activity with ABTS^•+^ compared to the DPPH^•^ assay [28].

According to the Mikucka et al., [23] in all distillery stillage extracts the main phenolic acid was *p*-coumaric acid and the ABTS^•+^ assay indicated significantly higher antioxidant activity than the DPPH^•^ assay. Moreover, the main phenolic acid in all our blackberry pomace extracts is *p*-coumaric acid, which may influence the higher antioxidant activity in the ABTS^•+^ assay. According to the Sodeinde et al. [30], the non-significant and low DPPH^•^ assay results in the extracts may be related to the low content of flavonoids and/or tannins in the extracts. Other researchers [31] have reported highly significant (*p* < 0.05) correlations of DPPH^•^ assay results with the flavonoid quercetin and quercetin derivatives, while our results show low amounts of this flavonoid in extracts (Table 2).

According to Piasecka et al. [26], blackberry pomace extracts, obtained under optimal UAE conditions (an ultrasound power—290 W, temperature—44 °C, extraction time—30 min), have 1712 mg 100 g^−1^ of TPC, 117.4 mg 100 g^−1^ of TAC and 109.21 μmol TE g^−1^ ABTS^•+^ antioxidant capacity, expressed as dry weight of extracts. Santos et al. [24] reported that blackberry pomace extract, obtained with UAE, has 5236 mg 100 g^−1^ of dry solid TPC and 139 mg 100 g^−1^ of dry solid of TAC. These results are consistent with those reported by Machado et al. [8] in blackberry pomace UAE extracts; TPC—5280 mg 100 g^−1^ of dry residue, TAC—237 mg 100 g^−1^ of dry residue, and an antioxidant capacity of DPPH^•^ radical—49.50 µmol TE g^−1^ of dry residue. In contrast to this finding, our research results showed lower TPC and TAC, probably due to the use of a low ethanol concentration (30%) for UAE extraction. On the other hand, low concentrations of ethanol and water can easily penetrate into cells, while high concentrations of ethanol can denature proteins, which inhibits the solubility of biologically active substances and affects the extraction rate [22]. According to the Jazic et al. [32], different extraction methods and conditions can affect the amount of polyphenols in the extracts and consequently the bioactivity of the extracts. Our obtained results show that it is also affected by the cultivar and the interaction between the cultivar and the extraction method.

### 2.2. Phenolic Profile of Blackberry Pomace Extracts

Blackberry pomace extracts from three different cultivars and extraction methods contained fourteen polyphenols. Variations in the amount of polyphenols were observed in the blackberry pomace extracts. The accumulation of specific metabolites, such as flavanols (catechin) and flavonols (kaemferol and quercetin) may be related to cultivar differences [33]. The results of the phenolic profile of blackberry pomace extracts are in agreement with those reported for blackberry pomace [4]. The main compound in the flavonoid group was epigallocatechin, the main compound in phenolic acid group was *p*-coumaric acid (Figure S1), and the main compound in anthocyanin group was cyanidin-3-*O*-glucoside (Figure S2), however, the amount of these compounds varied depending on the cultivar and extraction method

Epigallocatechin ranged from 8.183 mg mL^−1^ of extract in the MAE+UAE extract of cultivar ‘Brzezina’ to 0.244 mg mL^−1^ extract in MAE extract of cultivar ‘Polar’. The data show the general trend in the extraction efficiency of epigallocatechin and catechin for the ‘Orkan’ and ‘Brzezina’ cultivars in the MAE+UAE extract. Epigallocatechin and catechin are known for their antioxidant and anti-inflammatory properties [34].

Catechin ranges from 2.146 mg mL^−1^ in the MAE+UAE extract of the ‘Orkan’ cultivar to 0.044 mg mL^−1^ in the UAE extract of the ‘Orkan’ cultivar. Catechin is abundant, particularly in the skins of fruits and seeds. It acts as a precursor to condensed tannins and is similar to procyanidins. In addition to its function in disease prevention, it is considered a safe phytochemical for use in food applications [35]. The research results show that catechin in all cultivars correlated significantly positively with the ABTS^•+^ assay (Table 1 and Table 2) in MAE extract (r = 0.793, *p* < 0.05), in UAE extract (r = 0.715, *p* < 0.05) and in MAE+UAE extract (r = 0.701, *p* < 0.05).

Kaempferol ranges from 0.136 mg mL^−1^ in the UAE extract of the ‘Orkan’ cultivar to 0.004 mg mL^−1^ in the MAE extract of the same cultivar. The data show the general trend in the extraction efficiency of kaempferol for all cultivars in the UAE extract, whereas, the lowest amounts of this compound were found in the MAE extract of all cultivars. According to Yi Ling Yeong et al. [36], the extraction yield of certain types of flavonoids, including kaempferol, was higher using the UAE method than the MAE method, the author claims that this is due to the degradation of the active component that occurs.

The data show that there is a common tendency between ‘Polar’ and ‘Orkan’ cultivars that statistically significantly higher *p*-coumaric acid content is released in the MAE extract. A different extraction effect was observed in the pomace extract of the ‘Brzezina’ cultivar. The *p*-coumaric acid ranged from 0.776 mg mL^−1^ in the UAE extract of the ‘Brzezina’ cultivar to 0.009 mg mL^−1^ in the MAE extract of the same cultivar. Ellagic acid is a natural polyphenol with antioxidant and anti-inflammatory activities found in many fruits and vegetables [37]. The results show the general trend in the extraction efficiency of the ellagic acid for the all cultivars in MAE+UAE extract. Our research data show that ellagic acid correlates with the ABTS^•+^ assay (Table 1) due to its free radical scavenging properties, which are similar to those of chlorogenic acid [38].

Chlorogenic acid has strong radical scavenging properties that significantly enhance the total antioxidant potential of various plant extracts, such as those derived from blackberry pomace [39,40]. It has a significant positive relationship with the ABTS^•+^ assay results due to the numerous hydroxyl groups that can stabilise free radicals [40,41]. Moreover, the antioxidant potential of blackberry pomace extracts may be further enhanced by the synergistic effects of ellagic acid and chlorogenic acid. These acids may interact and possibly enhance each others effects when works boths, resulting in a stronger scavenging effect against ABTS^•+^ radicals [38,42]. A positive strong correlation was found between the ABTS^•+^ assay and two phenolic acids: chlorogenic (r = 0.924, *p* < 0.05) and ellagic (r = 0.754, *p* < 0.05) obtained from UAE extracts.

The other group of phenolic antioxidants found in blackberry pomace extracts are anthocyanins. The main anthocyanins in *R. idaeus* and *R. fruticosus* are derivatives of cyanidins, which are mostly present in unacylated form [43]. Cyanidin*-3*-*O*-glucoside ranged from 2.99 mg mL^−1^ in the MAE+UAE extract of cultivar ‘Brzezina’ to 1.76 mg mL^−1^ in the UAE extract of cultivar ‘Polar’. It is also important to note that cyanidin-3-*O*-glucoside and cyanidin-3-*O*-rutinoside in all cultivars correlated significantly negatively with the ABTS^•+^ assay in MAE extracts (r= −0.783 and r= −0.693, respectively then *p* < 0.05) and in UAE extracts (r= −0.839 and r= −0.713, respectively then *p* < 0.05). Studies show that cyanidin-3-*O*-glucoside and cyanidin-3-*O*-rutinoside have strong antioxidant properties, but their efficacy varies depending on the concentration and the presence of other compounds in the extracts [6]. According to Gong et al. [44], anthocyanins can negatively correlate with the ABTS^•+^ assay because these anthocyanins (cyanidin-3-*O*-glucoside and cyanidin-3-*O*-rutinoside) may interact with other phenolic compounds in the extract and their increasing concentration creates a complex interaction that affects the overall antioxidant capacity of the extract [44].

According to Salah-Eldin et al. [45], the dominant phenolic compound in UAE blackberry extract is gallic acid (46.396 mg 100 g^−1^ of extract). According to Kurek et al. [46], blackberry pomace ethanolic MAE extracts analysed by HPLC have 13.90 mg g^−1^ of anthocyanins, 1.49 mg g^−1^ of flavonoid glycosides and 1.34 mg g^−1^ of phenolic acids expressed as mg g^−1^ of pomace. Gong ES et al. [44], reported that ferulic acid, ellagic acid, kaempferol-O-hexoside and gallic acid were the major phenolic compounds in blackberry extracts of different cultivars and the differences in the samples tested were influenced by several factors such as: cultivar and extraction method [44]. Other researchers [47] reported that protocatechuic acid (3.36–35.18 mg g^−1^) and gallic acid (9.57–31.98 mg g^−1^) were the main phenolic compounds in pomace extracts of blackberry cultivars ‘Cacanska bestrna’ and ‘Thornfree’. Blackberry pomace extracts were identified to consist of phenolic acids (gallic, protocatechuic, caffeic, syringic, ellagic, syringic, vanillic, and synapic acids) and flavonoids (catechin, epicatechin, rutin, and myricetin) [32].

Summarising the results of the phenolic profile, it was found that the most efficient method to extract total flavonoids (12.948 mg mL^−1^ of extract) and total anthocyanins (5.45 mg mL^−1^ of extract) was MAE + UAE extraction, and the cultivar extract in which the highest total amounts were determined was ‘Brzezina’, while the most efficient method to extract total phenolic acids (0.990 mg mL^−1^ of extract) was UAE extraction method also from ‘Brzezina’ cultivar extract. ANOVA analysis showed a significant (*p* < 0.05) effect of cultivar on phenolic compounds (catechin, chlorogenic acid, epigallocatechin gallate, quercetin*-3*-*O*-rutinoside, kaempferol-3-*O*-glucoside, myricetin, quercetin, kaempferol, cyanidin-3-*O*-glucoside, cyanidin-3-*O*-rutinoside, ellagic acid) depending on the cultivar (Table S1). However, statistical analysis showed that cultivar had no effect on epigallocatechin (0.5802), *p*-coumaric acid (0.4078) and quercetin-3-*O*-glucoside (0.6576) content in the extracts. According to Suhaimy et al. [48], all these three compounds belong to flavonoid-based compounds and their synergistic effect is characterised by antiulcer activity.

Extraction method has no effect on myricetin content (0.1135) and the combined effect of both has no effect on cyanidin-3-*O*-rutinoside (0.1197), but analysis of other phenolic compounds showed a significant (*p* < 0.05) influence of cultivar, extraction method and their combined effect (Table S1).

### 2.3. Organic Acids and Sugars Profile of Blackberry Pomace Extracts

The content of sugars and organic acids, as well as the sugar/acid ratio, are important compositional parameters that influence the sensory properties of berry extracts [49]. Sugars are also essential for the synthesis of various compounds involved in antioxidant protection [49]. Although organic acids are less abundant than sugars, they have a greater influence on flavour because their presence makes it difficult to detect sweetness as acidity increases [50].

In addition to the blackberry cultivar, the extraction techniques used—such as MAE, UAE, and MAE+UAE—have a major influence on the amount of organic acids and sugars in blackberry pomace extracts. Each of these extraction methods can effectively influence the separation of sugars and organic acids from the pomace. Higher concentrations of sugars usually indicate sweetness, and the sugar/acid ratio is a key factor in the consumption of blackberry pomace products [51]. The highest content of total organic acids was found in the MAE extract of the ‘Polar’ cultivar (9.84 mg mL^−1^), while the lowest result was found in the MAE extract of the ‘Orkan’ cultivar (7.47 mg mL^−1^). The predominant organic acids, and perhaps the most important, were dehydroascorbic acid, citric acid, and malic acid (Figure S3).

The sum of ascorbic acid and dehydroascorbic acid is the total amount of vitamin C amount, which was higher in the MAE+UAE extract of the cultivar ‘Polar’ (2.79 mg mL^−1^); a very similar result was obtained in the MAE extract of the cultivar ‘Brzezina’ (2.78 mg mL^−1^); and the lowest amount was also in the MAE extract of the cultivar ‘Orkan’ (2.03 mg mL^−1^). The primary physiologically active form of vitamin C is ascorbic acid (AA), which can be reversibly oxidised to form dehydroascorbic acid (DHA) [52]. The AA can be oxidised to DHA during the extraction. Compared to AA, DHA is easier to transfer and more readily absorbed into plant membranes [53]. The acidity of berries and their products is caused by malic acid [54]. The highest malic acid content was found in the UAE extract of the ‘Polar’ and ‘Brzezina’ cultivars (1.98 and 2.26 mg mL^−1^, respectively), and in the MAE+UAE extract of the ‘Orkan’ cultivar (2.23 mg mL^−1^), while the lowest values were obtained in the MAE extracts of the cultivars ‘Polar’ and ‘Orkan’ (1.47 and 1.54 mg mL^−1^, respectively) and in the MAE+UAE extract of the cultivar ‘Brzezina’ (1.55 mg mL^−1^).

Sugar content varies according to the extraction method and the cultivar (Table 3). The highest total sugar content was found in the MAE+UAE extract of the cultivar ‘Orkan’ (11.71 mg mL^−1^), while the lowest was observed in the UAE extract of the cultivar ‘Polar’ (4.81 mg mL^−1^); these results differ more than two times. According to Mikulič-Petkovšek et al. [55], the predominant sugars in blackberry are glucose and fructose (Figure S4). The same predominant sugars were identified by Čechovičienė et al. [4] in pomace and in this study in extracts. According to Davidson, M et al. [56], the ultrasound-enzyme-assisted extraction method can significantly influence the sugar content in blackberry pomace extract compared to the conventional extraction method. Microwave heating has fewer negative effects on bioactive compounds than traditional conventional methods, with rapid increases in volume temperature without surface overheating, improved process control, and increased energy efficiency [57].

Analysing the results of organic acids and sugars, it was found that the most efficient method to extract total organic acids (9.84 mg mL^−1^ of extract) was MAE extraction, and the cultivar extract in which the highest total amounts were determined is ‘Polar’, while the most efficient method to extract total sugars (11.71 mg mL^−1^ of extract) was the MAE+UAE extraction method from the extract of the cultivar ‘Orkan’. A very similar result was also found in this cultivar using the UAE extraction method—9.48 mg mL^−1^ of extract.

### 2.4. Effects of Extracts on Cancer Cells

Plants enrich the human diet and can be widely used in the treatment of various diseases and play an important role in food and medicine [58]. Foods rich in polyphenols can reduce the risk of developing a number of diseases [59]. The effects of different cultivars, extraction methods, and different concentrations of extracts on the viability of the Caco-2 and CCD-18Co cell lines were analysed.

**Figure 1:**
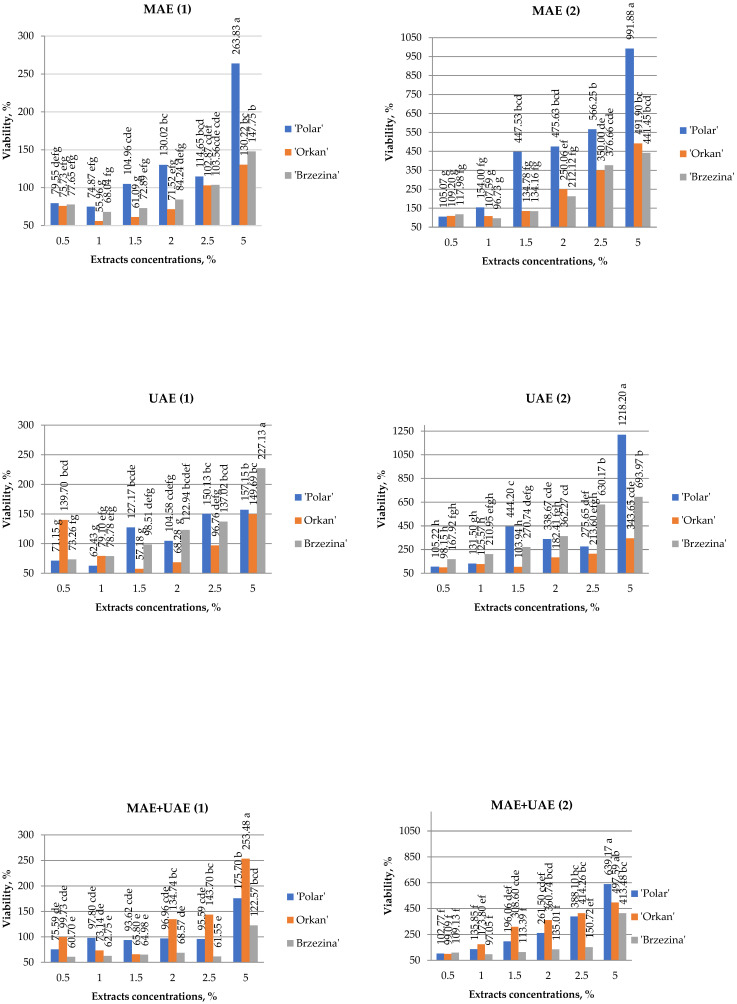
Influence of blackberry pomace extracts in Caco-2 colorectal adenocarcinoma cell line (1) and CCD-18Co normal colon fibroblasts (2) after 24 h incubation. Different lowercase letters indicate statistically significant differences (*p* < 0.05).

The results showed that the MAE extract of the ‘Orkan’ cultivar at 1%, 1.5%, and 2% concentrations most inhibited the growth of Caco-2 cells, while all concentrations of MAE extracts of the ‘Polar’ cultivar least inhibited the growth of Caco-2 cells (Figure 1). After evaluating the effect of the MAE extract of the ‘Brzezina’ cultivar, the growth of CCD-18Co cells was most inhibited when its concentrations were 1%, 1.5%, and 2%, while the fastest growth was stimulated by the ‘Polar’ cultivar and the highest viability was found in the 5% extract (991.88%) of the ‘Polar’ cultivar. As a result, we see a general trend wherein the MAE extract of the ‘Polar’ cultivar was the most favourable for the growth and proliferation of both cells (Caco-2 and CCD-18Co) (Figure 1). Moreover, the highest amount of organic acids (Table 3) was determined in the MAE extract of this cultivar, and other authors claim that a higher amount of organic acids can lead to the growth, migration, and metastasis of cancer cells [60].

Evaluating the efficacy of UAE extracts (Figure 1) against Caco-2 cells, it is clear that the ‘Orkan’ cultivar at 1.5%, 2%, and 2.5% concentrations of extract most inhibited the growth of Caco-2 cells, but the 0.5% concentration strongly accelerated their growth (139.70%); the viability was similar with a 5% concentration (149.69%). The efficacy of UAE extract (Figure 1) against CCD-18Co cells was evaluated, and the results show that the ‘Orkan’ cultivar at all concentrations most inhibited the growth of CCD-18Co cells. The difference with other cultivars is most evident at the 5% concentration (Figure 1); compared to the extract of the ‘Polar’ cultivar, the result for the ‘Orkan’ cultivar is 3.5 times lower, and compared to the extract of the ‘Brzezina’ cultivar, the result for the ‘Orkan’ cultivar is two times lower.

Evaluating the efficacy of MAE+UAE extracts, ‘Brzezina’ cultivar extracts most inhibited the viability of Caco-2 cells in all concentrations, while the ‘Orkan’ cultivar, which showed the most depressing effect in the previously mentioned extracts (MAE and UAE), in this MAE+UAE extract increased viability in the 0.5%, 2%, 2.5%, and 5% concentrations (Figure 1). In MAE+UAE extracts, extracts of the ‘Brzezina’ cultivar most inhibited the vitality of CCD-18Co cells at all concentrations, except for 0.5%, comparing the results with other cultivars (Figure 1). According to Morin et al. [61], blackberry volatile extracts inhibit cytokine secretion in LPS-stimulated macrophage cells, suggesting a broader anti-inflammatory potential that may also involve fibroblast cells such as CCD-18Co. The authors also emphasise that blackberry volatile extracts have stronger anti-inflammatory properties than phenolic extracts and that the volatile composition may be more important than the total volatile content [61].

After evaluating all the results of both Caco-2 and CCD-18Co cells, it is clear that a higher concentration (5%) increases the growth of cancer cells, so the recommended concentration that reduces the growth of these cells is between 1% and 2.5%, depending on the cultivar. When considering the viability of Caco-2 cells, their growth was best inhibited by MAE and UAE extracts of the ‘Orkan’ cultivar at concentrations of 1%, 1.5%, 2%, and 2.5%. However, Alzaid et al. [62] found no effect of berry extract treatment on Caco-2 cell viability (cells were treated with berry extract at a 0.125% concentration for 16 h, berry extracts from blueberry, bilberry, cranberry, elderberry, raspberry seed, and strawberry).

The cultivars ‘Orkan’ and ‘Brzezina’ show a general tendency to inhibit the viability of both cells the most, which may be due to the high content of individual flavonoids and anthocyanins in these cultivars (Table 2). In addition, the effect of MAE+UAE extract of the ‘Brzezina’ cultivar showed a general tendency to decrease the viability of both cancer cells (Caco-2 and CCD-18Co); this effect could be attributed to the fact that the ‘Brzezina’ cultivar MAE+UAE extract (Figure 1) had the highest amount of ellagic acid (Table 2), which is a strong antioxidant that acts against various cancer cells including breast [63] and colon cancer cells [64]. According to the Figueiras Abdala et al. [65], flavonoids and anthocyanins from blackberry UAE extracts can protect human cells from oxidative stress. Pap et al. [66] studied a number of berry species, including blackberry, and showed that blackberry pulp and seeds have anti-inflammatory, antihypertensive, antioxidant, and antihyperglycemic properties in vitro. According to the Tatar et al. [67], telomerase inhibition is a key mechanism by which blackberry exerts its anticancer effects in CRC cells.

## 3. Materials and Methods

### 3.1. Blackberry Pomace Sample Preparation

The blackberry cultivars ‘Polar’, ‘Orkan’, and ‘Brzezina’ were taken from the Joniškis region (56.30219045284591, 23.603429519328024) in Lithuania. The juice was extracted using a Stollar Commercial juicer (Stollar JES650, Riga, Latvia). The remaining pomace was freeze-dried in a lyophiliser ZIRBUS (sublimator 25, Harz, Germany) at −55 °C degrees, 48 h. Freeze-dried blackberry pomace was ground (Model Retsch ZM200, Haan, Germany) and stored in plastic bags at −38 °C degrees until the analyses.

### 3.2. Chemicals and Reagents

The ethanol of agricultural origin used for extraction was of analytical grade and was obtained from the MV Group (Kaunas, Lithuania). The reagents used in this study were as follows: acetonitrile (HPLC grade) Sigma-Aldrich Company (Poznan, Poland), acetone (HPLC grade) Sigma-Aldrich Company (Poznan, Poland), deionised water, ethyl acetate (HPLC grade) Merck (Poznan, Poland), polyphenols standards (epigallocatechin, catechin, chlorogenic acid, epigallocatechin gallate, *p*-coumaric, quercetin*-3*-*O*-rutinoside, kaempferol-3-*O*-glucoside, myricetin, quercetin, kaempferol, quercetin-3-*O*-glucoside, cyanidin-3-*O*-glucoside, cyanidin-3-*O*-rutinoside, 3-(4,5-dimethylthiazol-2-yl)-2,5-diphenyltetrazolium bromide)Sigma-Aldrich Company (Poznan, Poland), sulfuric acid 98% Merck Company (Poznan, Poland), DMEM medium GlutaMAX™ Supplement, fetal bovine serum (FBS), penicillin, and streptomycin (Gibco, TermoFisher Scientific, Waltham, MA, USA).

### 3.3. Extraction of Blackberry Pomace

The 150 g of blackberry pomace powder was mixed with 2000 mL of solvent ethanol:water ratio (30:70) [13]. The mixture was left in the dark at 5 °C for 24 h [68]. A microwave extractor (IDCO Microwave Industrial Solutions E200, Marseille, France) was used for the microwave extraction. The power level was 600 W for 30 min; the extraction temperature varied from 30 to 35 °C; and the extraction vessel pressure was 300 mbar. For ultrasound extraction, the power level was 250 W for 30 min [69] and the extraction temperature varied from 30 to 35 °C; the extraction vessel pressure was the same as for MAE. For microwave + ultrasound extraction, microwave extraction was performed first (power 600 W, time 30 min), followed by ultrasound extraction (power 250 W, time 30 min). The solvent concentration and other extraction parameters were chosen on the basis of various scientific studies, not only on blackberries, but also on the extraction of polyphenols from different matrices in general, selecting the optimal conditions for extraction [12,13,70,71]. The extracts were filtered with a Whatman filter, then the liquid extract was concentrated on a rotary evaporator at 50 °C until approximately 95% of the extraction solvent had been removed and stored at −28 °C until analysis.

### 3.4. Analysis of Total Polyphenolic Compounds (TPC)

The TPC was determined according to Bobinaite et al. [43]. Briefly, 1 g of blackberry extract was mixed with 40 mL of solvent (70% ethanol). The extracts were homogenised with a homogeniser (model VDI 25 s40, Germany) for 60 s each and left in the dark for 24 h, after which they were filtered with Whatman paper (retention 8–12 µm). Then, 1 mL of extract was mixed with 5 mL of Folin–Ciocalteu’s reagent (1:10 Folin:water) and after 60 s with 4 mL of Na_2_CO_3_ (7.5%) and then placed in the dark. After 60 min, the absorbance was measured using a Spectro UVD-3200 spectrophotometer (Spectro UV-VIS Double Beam PC, Labomed, Los Angeles, CA, USA) at a wavelength of 765 nm. The TPC was determined from the gallic acid calibration curve and expressed as mg per 100 g^−1^ soluble solids of extracts.

### 3.5. Analysis of the Total Anthocyanin Content (TAC)

The TAC was determined by the pH differential method [72]. Briefly, 0.2 g of extract was extracted with 10 mL of acidified ethanol (70%) and HCl (0.5%) in an 85:15 ratio. The extracts were allowed to stand in the dark for 24 h, after which they were filtered using Whatman paper (retention 8–12 μm). Then, 1 mL of filtered extract was mixed with 9 mL (pH1) solution of 0.025 M potassium chloride and 9 mL (pH4) solution of 0.4 M sodium acetate, both pH1 and pH4 separately, and left in the dark. After 30 min, the absorbance was measured using a Spectro UVD-3200 spectrophotometer (Spectro UV-VIS DoubleBeam PC, Labomed, USA) at wavelengths of 520 nm and 700 nm. The results were expressed as mg of cyanidin-3-*O*-glucoside equivalents per 100 g^−1^ soluble solids of extracts.

### 3.6. Analysis of the Total Flavonoid Content (TFC)

The TFC was analysed using the aluminium chloride colorimetric method. Briefly, 1 g of blackberry extract was mixed with 10 mL of ethanol (75%) and left for 60 min in an automatic shaker (Heidolph Vibramax 100, 31W, Tiefenbach, Germany) (1200 rpm), after which it was filtered using Whatman paper (retention 8–12 µm). Briefly, 1 mL of the filtered extract was mixed with 10 mL of aluminium chloride solution (2% *m*/*v*), 2 mL of ethanol (96%), and 1 mL of 1 M sodium acetate and left in the dark. After 40 min, the absorbance was measured using a Spectro UVD-3200 spectrophotometer (Spectro UV-VIS Double Beam PC, Labomed, USA) at a wavelength of 420 nm [73]. The results were expressed as mg quercetin per 100 g^−1^ soluble solids of extracts.

### 3.7. Analysis of Individual Polyphenols

HPLC was used to identify and quantify individual polyphenols [74]. After mixing 1 mL of extract with 80% methanol, it was extracted in an ultrasonic bath for 10 min at 6000 Hz and 30 °C. The samples were then centrifuged at 6000 rpm for 10 min at 0 °C. A Fusion RP-80 A column, with a grain particle size of 2.5 (250 × 4.6 mm, Phenomenex, Warsaw, Poland) was filled with 100 µL of the mixture. A UV-Vis detector (SPD-20AV), column oven (CTO-20AC), autosampler (SIL-20AC), controller unit (CMB-20A), and two pumps (LC-20AD) were part of the HPLC system (Shimadzu, USA Manufacturing Inc., Canby, OR, USA). The gradient phase, consisting of acetonitrile and water with phosphoric acid (pH 3.0), was used at a flow rate of 1 mL min^−1^. Phase A used 10% acetonitirile, 90% dejonised water and Phase B used 55% acetonitrile and 45% dejonised water. The time phases were as follows: 1.00–22.99 min, phase A 95% and phase B 5%; 23.00–27.99 min phase A 50% and phase B 50%; 28.00–28.99 min phase A 80% and phase B 20%; and 29.00–38.00 min phase A 50% and phase B 50%, last 39.00–42.00 min phase A 95% and phase B 5%. The analysis time was 42 min, the detection wavelength for flavonoids was 360 nm, and the detection wavelength for phenolic acids was 250 nm. Polyphenols were identified on the basis of the retention time, using external standards. The results were expressed as mg mL ^−1^ of extract.

### 3.8. Analysis of Organic Acids and Sugars

The organic acid and sugar content of blackberry pomace hydrophilic extracts was determined using the HPLC method [75]. Briefly, 0.5 mL of extract was collected in plastic test tubes, and diluted 10 times with water (ratio 0.5:4.5). The samples were centrifuged for 10 min in a centrifuge (5000 rpm). Then, 1.5 mL of each sample was filtered through 0.22 μm PES syringe filters for HPLC vials. Standard curves were generated and identified in the chromatogram on the basis of the retention time of the standard compounds. The parameters for the HPLC analysis were as follows: isocratic phase: 10 mM sulfuric acid, flow rate 0.5 mL/min; injection: 25 μL; column used: Aminex HPX-87H (300 × 7.8 mm, 9 μm, 8% cross linkage, pH 1–3); analysis time: 1 h 15 min; detection: 210 nm; and analysis temperature: 40 °C. Organic acids and sugars was expressed as mg mL ^−1^ of extract. The sum of all organic acids was defined as the total organic acid content and the sum of all sugars was defined as the total sugar content.

### 3.9. Assay for Antioxidant Activity ABTS^•+^ Discoloration Method

The antioxidant activity of blackberry pomace extracts was evaluated using the Trolox equivalent antioxidant capacity assay and the ABTS^•+^ discoloration method. A Spectro UVD-3200 (Spectro UV-VIS Double Beam PC, Labomed, USA) spectrophotometer was used to measure the decrease in absorbance at 734 nm after sequential extraction of fractions from 0.1 g of each sample using methanol and acetone (1:1, *v*/*v*) in an ultrasonic bath for one hour. The mixture was then filtered through Whatman paper (retention 8–12 µm) and 20 μL was combined with 2.0 mL of ABTS^•+^ solution. Trolox concentrations at a wavelength of 734 nm were used to calculate the antioxidant activity of the samples and a fixed inhibition time of 30 min was used. The antioxidant activities were expressed as µmol TE g^−1^ soluble solids of extracts [76].

### 3.10. Analysis of DPPH^•^ Radical Scavenging Activity (DPPH^•^-RSA)

Extracts from blackberry pomace were tested for their ability to scavenge radicals. The process is based on the fact that antioxidant compounds cause the stable DPPH^•^ radical to turn yellow. Briefly, 20 μL of the extract obtained was combined with 2 mL of DPPH^•^ ethanolic solution (the procedure is the same as for the ABTS^•+^ discoloration method). After 30 min, the absorbance was measured using a spectrometer Spectro UVD-3200 (Spectro UV-VIS Double Beam PC, Labomed, USA) at a wavelength of 515 nm. DPPH^•^ radical scavenging activity was expressed as Trolox equivalents μmol TE g^−1^ soluble solids of extracts [76].

### 3.11. Human Cell Lines

The effect of the extracts was studied in the Caco-2 colorectal adenocarcinoma cell line and CCD-18Co normal colon fibroblasts obtained from the American Type Culture Collection (ATCC, Manassas, VA, USA). The Caco-2 cell line was cultured in DMEM medium GlutaMAX™ Supplement (Gibco, TermoFisher Scientific) supplemented with 10% fetal bovine serum (FBS) (Gibco, TermoFisher Scientific), penicillin (100 U/mL) (Gibco, TermoFisher Scientific), and streptomycin (100 μg/mL) (Gibco, TermoFisher Scientific) at 37 °C in a humidified atmosphere of 5% CO_2_ in air. A fetal bovine serum (FBS) concentration of 10% is recommended by the ATCC. The CCD-18Co cell line was cultured in EMEM medium GlutaMAX™ Supplement (Gibco, TermoFisher Scientific) supplemented with 10% fetal bovine serum (FBS) (Gibco, TermoFisher Scientific), penicillin (100 U/mL) (Gibco, TermoFisher Scientific), and streptomycin (100 μg/mL) (Gibco, TermoFisher Scientific) at 37 °C in a humidified atmosphere of 5% CO_2_ in air. A fetal bovine serum (FBS) concentration of 10% is recommended by the ATCC. Cell viability in the two cell lines was assessed in the range of 0.5% to 5% extract concentrations. The incubation time was 24 h [77].

### 3.12. Estimation of Tested Extracts Cytotoxicity

The compounds were added to the cultured cells to give a final extract concentration in the range of 0.5% to 5%. Control cells were incubated without the test compounds. The extracts’ cytotoxicity was measured by using MTT assay. This is a method proposed by Carmichael using 3-(4,5-dimethylthiazol-2-yl)-2,5-diphenyltetrazolium bromide [78]. Caco-2 cells and CCD-18Co cells were seeding in a 96-well plate at a density of 1 × 10^4^ cells/well. Cells cultured for 24 h were treated with extracts. After 24 h, the cells were washed 3 times with PBS and subsequently incubated with 10 µL of MTT solution (5 mg/mL in PBS) for 2 h at 37 °C in 5% CO_2_ in an incubator. Subsequently, 100 µL of DMSO was added and cells were incubated in the dark for the next 2 h. The absorbance was measured at 560 nm in a microplate plate reader GloMax^®^-Multi Microplate Multimode Reader. The viability of the cells was calculated as a percentage of the control cells, incubated without the tested compound [77].

### 3.13. Statistical Analysis

Data are expressed as means ± standard deviations for at least three independent measurements. Statistical analyses were performed using two-way analysis of variance (ANOVA). Fisher’s post hoc test was applied to assess significant differences then *p* < 0.05. Pearson’s correlation coefficient was calculated to determine the relationship between the variables then *p* < 0.05.

## 4. Conclusions

During the extraction process, specific biologically active compounds were isolated and concentrated, increasing the potential for their use in a form other than pomace.

The main phenolics obtained in the extracts were epigallocatechin, *p*-coumaric acid and cyanidin-3-*O*-glucoside. The most efficient method to extract organic acids was MAE extraction of the cultivar ‘Polar’, while the most efficient method to extract sugars was MAE+UAE extraction of the cultivar ‘Orkan’. The main organic acids in the extracts were dehydroascorbic acid, citric acid, and malic acid. The main sugars in the blackberry extracts were glucose and fructose.

MAE+UAE extracts of the ‘Brzezina’ cultivar most inhibited the viability of Caco-2 cells at all concentrations, and of CCD-18Co cells at all concentrations, except for 0.5%.

Overall, the most valuable cultivar for extractions is ‘Brzezina’, and the most effective method for excluding biologically active compounds and for inhibiting the viability of cancer cells is the combined MAE+UAE extraction method.

Blackberry pomace extracts, due to their rich polyphenolic profile, can be used as an excipient in pharmacy and medicine or to impart functional properties to food products (beverages, bakery products, dairy products, sweets, etc.).

## Figures and Tables

**Table 1:** Total phenolic content (TPC), total anthocyanin content (TAC), total flavonoid content (TFC), and antioxidant activity of blackberry pomace extracts, soluble solids of extracts.

Cultivar	‘Polar’			’Orkan’			‘Brzezina’			*p* Value		
Name/Extraction Method	MAE	UAE	MAE+UAE	MAE	UAE	MAE+UAE	MAE	UAE	MAE+UAE	Cultivar	Extraction Method	Cultivar x Extraction Method
	mg 100 g^−1^											
TPC	3274.10 ± 400.92 bc	3535.14 ± 85.82 ab	3376.97 ± 396.11 abc	3327.59 ± 192.04 abc	3694.25 ± 51.23 a	2242.69 ± 98.27 d	3438.41 ± 231.89 abc	2032.75 ± 188.10 d	3072.96 ± 229.82 c	0.0001	0.0001	0.0001
TAC	78.56 ± 5.69 e	115.54 ± 4.49 b	93.15 ± 4.53 cd	97.22 ± 2.28 c	111.30 ± 5.64 b	85.46 ± 2.97 de	129.91 ± 3.60 a	91.66 ± 2.0 cd	88.53 ± 7.16 d	0.0001	0.0001	0.0001
TFC	187.39 ± 3.54 d	239.88 ± 41.55 a	185.62 ± 0.75 d	197.64 ± 3.06 cd	200.11 ± 0.92 bcd	226.08 ± 13.47 ab	187.79 ± 1.55 d	93.53 ± 3.50 e	219.14 ± 21.51 abc	0.0001	0.0001	0.0001
	µmol TE g^−1^											
DPPH^•^	71.46 ± 12.33 a	66.73 ± 11.26 a	77.43 ± 15.35 a	84.86 ± 17.58 a	85.86 ± 14.37 a	76.43 ± 10.51 a	82.00 ± 13.90 a	74.33 ± 12.70 a	84.53 ± 15.12 a	0.2600	0.7989	0.7068
ABTS^•+^	286.16 ± 36.35 ab	303.80 ± 17.96 a	223.70 ± 28.93 de	195.23 ± 21.45 ef	171.16 ± 7.14 f	261.63 ± 29.54 bcd	236.00 ± 22.90 cde	270.85 ± 13.63 abc	197.68 ± 23.66 ef	0.0001	0.2048	0.0001

Values expressed as mean ± standard deviation (SD). In each row, different lowercase letters indicate statistically significant differences (*p* < 0.05).

**Table 2:** Phenolic profile of blackberry pomace extracts obtained by different extraction methods, mg mL^−1^ of extract.

Cultivar	‘Polar‘			‘Orkan’			‘Brzezina’		
Name/Extraction Method	MAE	UAE	MAE+UAE	MAE	UAE	MAE+UAE	MAE	UAE	MAE+UAE
*Flavonoids*									
Epigallocatechin	0.244 ± 0.008 d	5.241 ± 0.379 b	3.715 ± 0.545 c	0.365 ± 0.053 d	0.575 ± 0.029 d	7.834 ± 0.124 a	0.361 ± 0.008 d	0.308 ± 0.009 d	8.183 ± 0.599 a
Catechin	0.560 ± 0.014 c	1.096 ± 0.040 b	0.546 ± 0.050 c	0.192 ± 0.008 e	0.044 ± 0.001 f	2.146 ± 0.073 a	0.469 ± 0.010 d	0.113 ± 0.006 f	0.529 ± 0.082 cd
Epigallocatechin gallate	0.005 ± 0.000 de	0.009 ± 0.000 a	0.005 ± 0.000 d	0.005 ± 0.001 de	0.007 ± 0.000 b	0.005 ± 0.000 d	0.007 ± 0.000 c	0.005 ± 0.000 e	0.006 ± 0.000 d
Quercetin-3-*O*-rutinoside	0.155 ± 0.007 cd	0.219 ± 0.005 a	0.019 ± 0.003 b	0.155 ± 0.001 cd	0.145 ± 0.010 de	0.134 ± 0.010 e	0.072 ± 0.007 f	0.049 ± 0.001 g	0.168 ± 0.015 c
Kaempferol-3-*O*-glucoside	0.024 ± 0.001 b	0.007 ± 0.001 d	0.013 ± 0.001 c	0.024 ± 0.000 a	0.006 ± 0.001 e	0.006 ± 0.000 e	0.009 ± 0.001 d	0.009 ± 0.001 d	0.006 ± 0.000 e
Myricetin	0.006 ± 0.000 de	0.007 ± 0.001 cde	0.006 ± 0.000 e	0.011 ± 0.000 a	0.007 ± 0.000 cde	0.008 ± 0.000 bc	0.003 ± 0.000 f	0.008 ± 0.001 b	0.007 ± 0.001 bcd
Quercetin	0.004 ± 0.000 b	0.003 ± 0.000 c	0.003 ± 0.000 c	0.003 ± 0.000 c	0.003 ± 0.000 cd	0.003 ± 0.000 c	0.005 ± 0.000 a	0.005 ± 0.001 a	0.003 ± 0.000 d
Kaempferol	0.005 ± 0.001 e	0.047 ± 0.004 c	0.035 ± 0.002 d	0.004 ± 0.000 e	0.136 ± 0.003 a	0.113 ± 0.007 b	0.005 ± 0.000 e	0.034 ± 0.001 d	0.032 ± 0.003 d
Quercetin-3-*O*-glucoside	0.011 ± 0.000 c	0.022 ± 0.002 a	0.011 ± 0.000 c	0.011 ± 0.000 c	0.016 ± 0.002 b	0.015 ± 0.004 b	0.015 ± 0.000 b	0.014 ± 0.001 bc	0.014 ± 0.002 bc
*Phenolic acids*									
Chlorogenic acid	0.070 ± 0.001 f	0.197 ± 0.007 b	0.240 ± 0.006 a	0.235 ± 0.006 a	0.083 ± 0.004 e	0.142 ± 0.003 d	0.047 ± 0.005 g	0.201 ± 0.010 b	0.155 ± 0.004 c
Ellagic acid	0.036 ± 0.001 c	0.035 ± 0.001 c	0.045 ± 0.001 a	0.036 ± 0.002 c	0.010 ± 0.001 e	0.041 ± 0.002 b	0.011 ± 0.001 de	0.013 ± 0.000 d	0.044 ± 0.003 ab
*p*-coumaric acid	0.638 ± 0.046 ab	0.321 ± 0.011 c	0.558 ± 0.001 b	0.505 ± 0.348 bc	0.339 ± 0.027 c	0.555 ± 0.013 b	0.009 **±** 0.000 d	0.776 ± 0.044 a	0.503 ± 0.005 bc
*Anthocyanins*									
Cyanidin-3-*O*-glucoside	2.18 ± 0.02 f	1.76 ± 0.01 g	2.52 ± 0.01 d	2.72 ± 0.05 b	2.65 ± 0.01 c	2.54 ± 0.00 d	2.62 ± 0.01 c	2.42 ± 0.01 e	2.99 ± 0.01 a
Cyanidin-3-*O*-rutinoside	1.81 ± 0.03 d	1.55 ± 0.06 e	2.04 ± 0.02 c	2.13 ± 0.03 bc	2.06 ± 0.04 bc	2.23 ± 0.31 b	2.17 ± 0.04 bc	2.01 ± 0.01 c	2.46 ± 0.01 a

Values expressed as mean ± standard deviation (SD). In each row, different lowercase letters indicate statistically significant differences (*p* < 0.05).

**Table 3:** Organic acids and sugars content in blackberry pomace extracts obtained by different extraction methods, mg mL^−1^ of extract.

Cultivar	‘Polar’			‘Orkan’			‘Brzezina’			*p* Value		
Name/Extraction Method	MAE	UAE	MAE+ UAE	MAE	UAE	MAE+UAE	MAE	UAE	MAE+UAE	Extraction Method	Cultivar	Extraction Method x Cultivar
*Organic acids*												
Dehydroascorbic acid	2.42 ± 0.04 c	2.49 ± 0.00 b	2.77 ± 0.02 a	2.01 ± 0.01 f	2.19 ± 0.00 e	2.31 ± 0.01 d	2.76 ± 0.11 a	2.28 ± 0.06 d	2.16 ± 0.00 e	0.0000	0.0000	0.000
L-ascorbic acid	0.03 ± 0.1 a	0.03 ± 0.00 a	0.02 ± 0.00 b	0.02 ± 0.00 b	0.04 ± 0.00 a	0.04 ± 0.00 a	0.02 ± 0.00 b	0.03 ± 0.01 a	0.02 ± 0.00 b	0.0040	0.0486	0.0017
Citric acid	5.92 ± 0.22 a	4.80 ± 0.04 b	4.56 ± 0.00 bc	3.90 ± 0.00 e	4.43 ± 0.00 cd	4.56 ± 0.04 bc	4.25 ± 0.00 d	4.37 ± 0.49 cd	4.13 ± 0.00 de	0.0187	0.0000	0.0000
Malic acid	1.47 ± 0.06 i	1.98 ± 0.03 c	1.49 ± 0.02 gh	1.54 0.01 fg	2.18 ± 0.02 b	2.23 ± 0.03 ab	1.66 ± 0.00 d	2.26 ± 0.06 a	1.55 ± 0.00 e	0.0000	0.0000	0.000
Total organic acids content	9.84 ± 0.16 a	9.31 ± 0.08 b	8.85 ± 0.05 cd	7.48 ± 0.03 f	8.85 ± 0.02 cd	9.14 ± 0.09 bc	8.69 ± 0.02 d	8.95 ± 0.51 cd	7.87 ± 0.01 e	0.0000	0.0002	0.0000
*Sugars*												
Glucose	5.80 ± 0.28 cd	4.55 ± 0.31 e	5.33 ± 0.05 de	6.36 ± 0.65 bc	7.15 ± 0.98 b	8.18 ± 0.95 a	6.83 ± 0.21 b	6.85 ± 0.11 b	5.57 ± 0.20 cd	0.7613	0.0000	0.0003
Fructose	1.62 ± 0.26 bc	0.26 ± 0.11 d	0.91± 0.09 cd	1.40 ± 0.96 bcd	2.33 ± 1.06 b	3.53 ± 1.34 a	2.17 ± 0.18 b	1.98 ± 0.20 bc	0.88 ± 0.07 cd	0.7092	0.0007	0.0015
Total sugar content	7.42 ± 0.44 cd	4.81 ± 0.22 e	6.24 ± 0.08 de	7.76 ± 1.60 bcd	9.48 ± 2.03 b	11.71 ± 2.30 a	9.00 ± 0.34 bc	8.84 ± 0.29 bc	6.45 ± 0.14 de	0.0000	0.7215	0.0006

Values expressed as mean ± standard deviation (SD). In each row, different lowercase letters indicate statistically significant differences (*p* < 0.05).

## Data Availability

The data are contained within the article.

## Supplementary Materials

The following supporting information can be downloaded at: https://www.mdpi.com/article/10.3390/plants14030384/s1, Table S1: *p* value for the phenolic profile of blackberry pomace extracts. Figure S1: Exemplary chromatogram of polyphenols identified in extract of blackberry pomace ultrasound-assisted extraction (UAE) ‘Polar’ cultivar; Figure S2: Exemplary chromatogram of anthocyanins identified in extract of blackberry pomace microwaved-assisted extraction (MAE), ‘Orkan’ cultivar; Figure S3: Exemplary chromatogram of organic acids identified in extract of blackberry pomace ultrasound-assisted extraction (UAE), ‘Polar’ cultivar; Figure S4: Exemplary chromatogram of sugars identified in extract of blackberry pomace ultrasound-assisted extraction (UAE), ‘Orkan’ cultivar.
