# Supplementary material for: Ultrasound and Microwave-Assisted Extraction of Blackberry (Rubus fruticosus L.) Pomace: Analysis of Chemical Properties and Anticancer Activity
Source: Plants (Basel). 2025 Jan 27;14(3):384. doi: 10.3390/plants14030384 (PMC11820506; doi:10.3390/plants14030384)
Supplement: Supplementary file 1 [file plants-14-00384-s001.zip › plants-3416677-supplementary.pdf]

Table S1. P value for the phenolic profile of blackberry pomace extracts.

Name	P value		
	Extraction method	Cultivar	Extraction method x Cultivar
	Flavonoids		
Epigallocatechin	0.0000	0.5802	0.0000
Catechin	0.0000	0.0000	0.0000
Epigallocatechin gallate	0.0000	0.0002	0.0000
Quercetin-3-O-rutinoside	0.0000	0.0000	0.0000
Kaempferol-3-O-glucoside	0.0000	0.0000	0.0000
Myricetin	0.1135	0.0000	0.0000
Quercetin	0.0000	0.0000	0.0000
Kaempferol	0.0000	0.0000	0.0000
Quercetin-3-O-glucoside	0.0000	0.6577	0.0001
	Phenolic acids		
Chlorogenic acid	0.0000	0.0000	0.0000
Ellagic acid	0.0000	0.0000	0.0000
p-coumaric	0.0392	0.4079	0.0000
	Anthocyanins		
Cyanidin-3-O-glucoside	0.0000	0.0000	0.0000
Cyanidin-3-O-rutinoside	0.0000	0.0000	0.1197

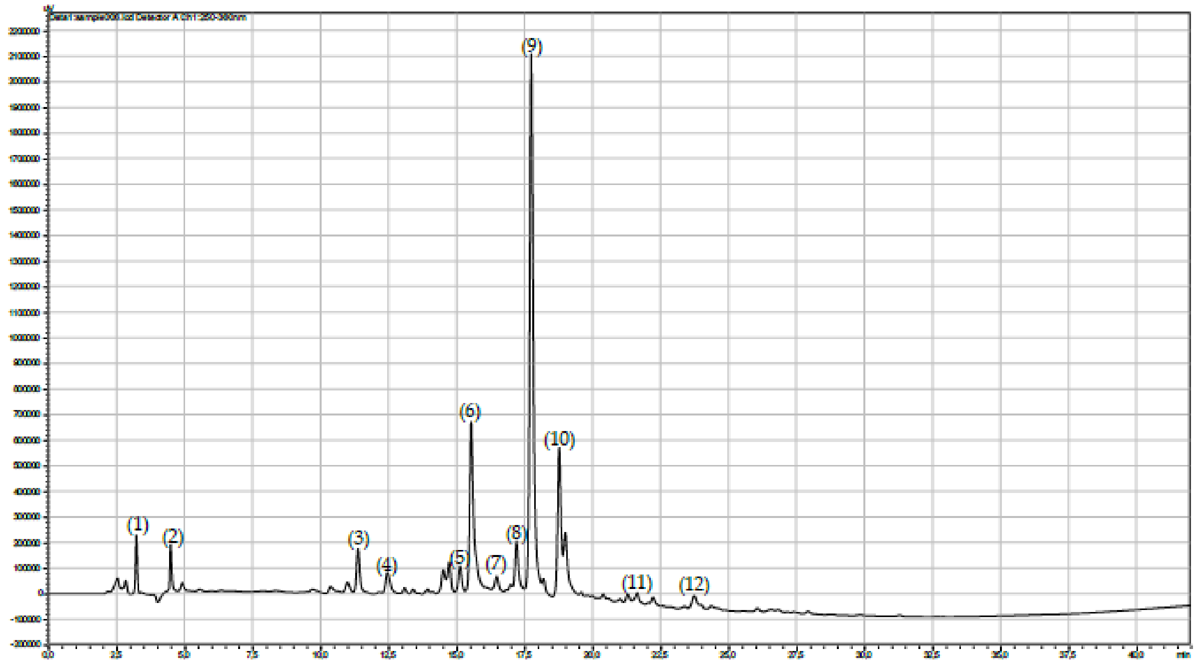

Figure S1. Exemplary chromatogram of polyphenols identified in extract of blackberry pomace ultrasound-assisted extraction (UAE) 'Polar' cultivar.

(1) *p*-coumaric acid, (2) chloragenic, (3) ellagic acid, (4) kaempferol, (5) quercetin-3-*O*-glucoside, (6) quercetin-3-*O*-rutinoside, (7) epigallocatechin gallate, (8) kaempferol-3-*O*-glucoside, (9) epigallocatechin, (10) catechin, (11) myrycetin, (12) quercetin.

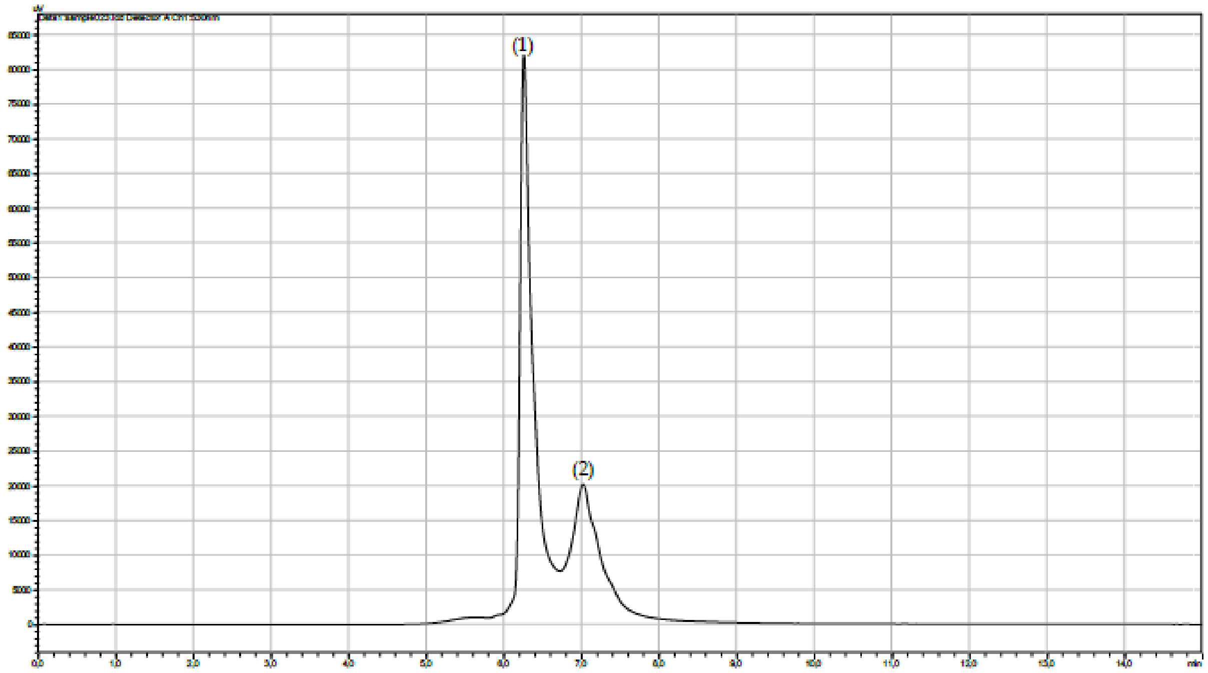

Figure S2. Exemplary chromatogram of anthocyanins identified in extract of blackberry pomace microwaved-assisted extraction (MAE), 'Orkan' cultivar.
(1) Cyanidin-3-*O*-glucoside, (2) Cyanidin-3-*O*-rutinoside.

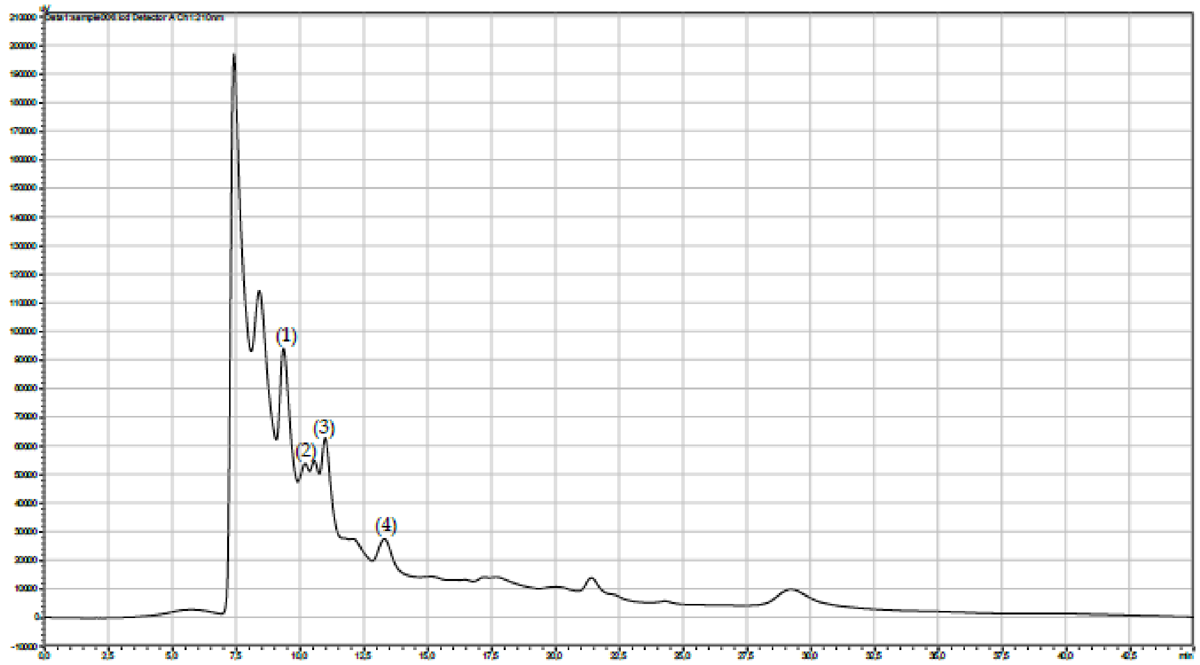

Figure S3. Exemplary chromatogram of organic acids identified in extract of blackberry pomace ultrasound-assisted extraction (UAE), 'Polar' cultivar.
(1) citric acid, (2) dehydroascorbic acid, (3) malic acid, (4) l-ascorbic acid.

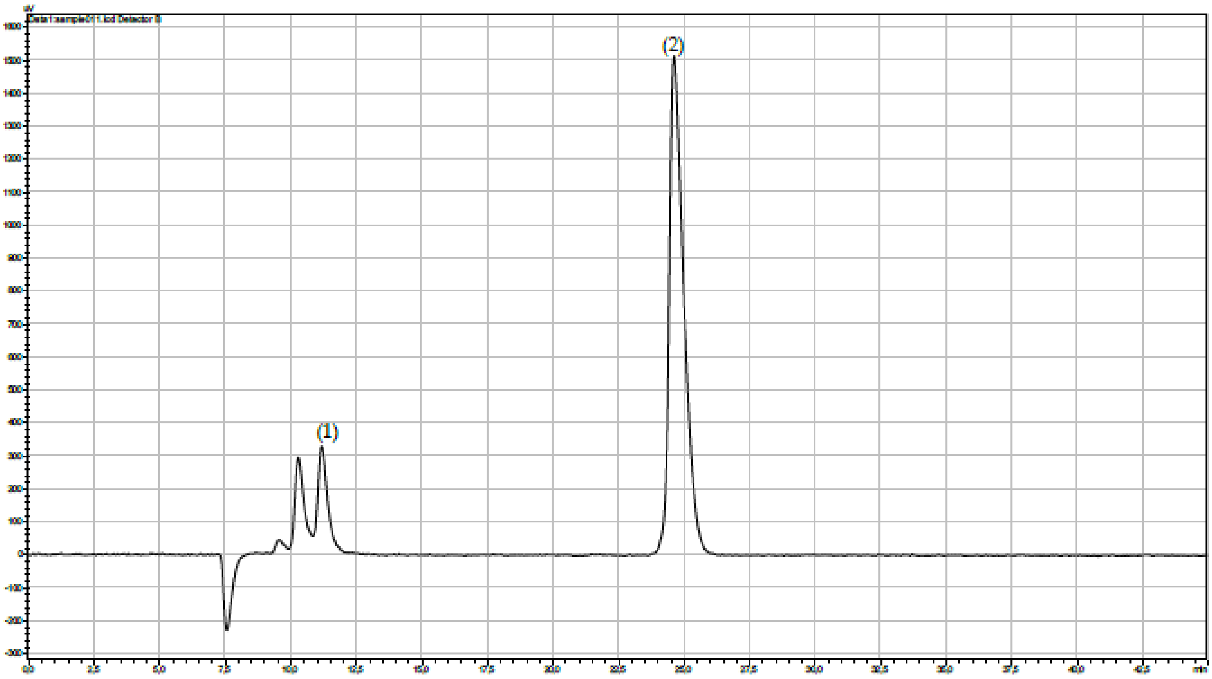

Figure S4. Exemplary chromatogram of sugars identified in extract of blackberry pomace ultrasound-assisted extraction (UAE), 'Orkan' cultivar.
(1) fructose,(2) glucose.
